# Pre-Treatment of Recombinant Mouse MFG-E8 Downregulates LPS-Induced TNF-α Production in Macrophages via STAT3-Mediated SOCS3 Activation

**DOI:** 10.1371/journal.pone.0027685

**Published:** 2011-11-15

**Authors:** Monowar Aziz, Asha Jacob, Akihisa Matsuda, Rongqian Wu, Mian Zhou, Weifeng Dong, Weng-Lang Yang, Ping Wang

**Affiliations:** 1 Center for Immunology & Inflammation, The Feinstein Institute for Medical Research, Manhasset, New York, United States of America; 2 Department of Surgery, North Shore University Hospital and Long Island Jewish Medical Center, Manhasset, New York, United States of America; University of Illinois at Chicago, United States of America

## Abstract

Milk fat globule-epidermal growth factor factor 8 (MFG-E8) regulates innate immune function by modulating cellular signaling, which is less understood. Herein, we aimed to investigate the direct anti-inflammatory role of MFG-E8 in macrophages by pre-treatment with recombinant murine MFG-E8 (rmMFG-E8) followed by stimulation with LPS in RAW264.7 cells and in peritoneal macrophages, isolated from wild-type (WT) or MFG-E8^−/−^ mice. RAW264.7 cells and mouse peritoneal macrophages treated with rmMFG-E8 significantly downregulated LPS-induced TNF-α mRNA by 25% and 24%, and protein levels by 29% and 23%, respectively (P<0.05). Conversely, peritoneal macrophages isolated from MFG-E8^−/−^ mice produced 28% higher levels of TNF-α, as compared to WT mice when treated with LPS. In *in vivo*, endotoxemia induced by intraperitoneal injection of LPS (5 mg/kg BW), at 4 h after induction, serum level of TNF-α was significantly higher in MFG-E8^−/−^ mice (837 pg/mL) than that of WT (570 pg/mL, P<0.05). To elucidate the direct anti-inflammatory effect of MFG-E8, we examined STAT3 and its target gene, SOCS3. Treatment with rmMGF-E8 significantly induced pSTAT3 and SOCS3 in macrophages. Similar results were observed in *in vivo* treatment of rmMFG-E8 in peritoneal cells and splenic tissues. Pre-treatment with rmMFG-E8 significantly reduced LPS-induced NF-κB p65 contents. These data clearly indicated that rmMFG-E8 upregulated SOCS3 which in turn interacted with NF-κB p65, facilitating negative regulation of TLR4 signaling for LPS-induced TNF-α production. Our findings strongly suggest that MFG-E8 is a direct anti-inflammatory molecule, and that it could be developed as a therapy in attenuating inflammation and tissue injury.

## Introduction

The glycoprotein, MFG-E8 is mainly secreted by mononuclear cells, and participates in the phagocytosis of apoptotic cells by tethering between phosphatidylserine (PS) on apoptotic cells and α_v_β_3_-integrin on phagocytes [1]. Detrimental autoimmune diseases are likely to be seen in MFG-E8^−/−^ mice due to the infiltration of apoptotic cells at the germinal centers of spleen [2], [3]. With the concept of removing the dead cells from various organs, MFG-E8 has proved to be an essential factor in controlling the progression of various inflammatory diseases [4], [5]. However, recent studies showed that MFG-E8-mediated potential therapeutic benefits in sepsis and intestinal injury were not only solely dependent on the enhanced clearance of apoptotic cells, but also rely on diverse cellular events to maintain the epithelial integrity and healing of the injured mucosa via protein kinase C (PKC) pathways [6]. Furthermore, MFG-E8-mediated upregulation of Akt, twist and MAP kinase signaling to perform several intra-cellular functions has also been reported [7], [8].

In mammals, Toll like receptor (TLR) 4 expressed in immune-reactive cells can efficiently recognize bacterial lipopolysaccharide (LPS) and trigger down-stream signaling via several adaptor molecules, e.g., myeloid differentiation factor 88 (MyD88), Toll/IL-1 receptor domain-containing adaptor (TIRAP)/MyD88-adaptor-like (Mal), IL-1 receptor-associated kinase (IRAK), and TNF receptor-associated factor 6 (TRAF6) to induce NF-κB, MAP kinases and JNK pathways. This in turn upregulates the expression of pro-inflammatory cytokines, e.g., tumor necrosis factor (TNF)-α [9], [10]. Uncontrolled activation of innate immune system by LPS may cause “endotoxin shock” [11], attributing a tightly controlled mechanism for its regulation. To maintain the fine-tuned balance of TLR4 pathway, several intracellular negative regulators, e.g., A20, IRAK-M, and Tollip are also become activated under LPS-treated conditions [12], [13].

Signal transducer and activator of transcription (STAT) 3 is known to play essential roles in maintaining the homeostatic balance between pro- and anti-inflammatory signals in immune competent cells induced by a wide ranges of cytokines, interleukin (IL)-5, 6, 10, interferon (IFN)-γ, epidermal growth factor (EGF), hepatocyte growth factor (HGF), hormones (prolactin, leptins) and other factors, leukemia inhibitory factor (LIF), bone morphogenetic protein (BMP)-2 [14]–[16]. Although there are seven members of STAT proteins available in cellular systems, the role of STAT3 is widely described in the field of innate-immune system induced by those cytokines and growth factors. The activation of STAT3 by various ligands occurs by binding to their respective receptors and receptor-associated kinases. The binding of IL-6 family of cytokines to the gp130 receptor triggers STAT3 phosphorylation by janus kinase (JAK) 2 [17]. Epidermal growth factor receptor and certain other receptor tyrosine kinases, such as c-MET, phosphorylate STAT3 in response to their ligands [18]. STAT3 is also a target of the c-Src non-receptor tyrosine kinase induced by various ligands [19]. Since STAT3-deficient mice are lethal [20], studies in conditional knock-out mice containing STAT3-deficient monocytes and neutrophils showed a reduced survival from endotoxic shock [21], [22]. An intriguing feature of STAT3 signaling lies with the fact that its function is firmly controlled by the STAT-induced STAT inhibitor (SSI) family of proteins, that are also known as suppressor of cytokine signaling (SOCS). In mammals, eight members of the SOCS proteins (SOCS1-7 and CIS) have been reported [23]. SOCS1 and 3 possess a kinase-inhibitory region (KIR) domain that is critical for inhibition of kinase activity [24]. In addition, over-expression studies have shown that both SOCS1 and 3 exhibit negative regulatory effects on TLR4 signaling [25]. Recent reports demonstrated that LPS-induced pro-inflammatory cytokine production and NF-κB activation are inhibited in cells over-expressing SOCS proteins [25], [26]. Several adaptor molecules that bridge between TLR4 and NF-κB, e.g., MAL/TIRAP, IRAK1 are the target of SOCS for ubiquitination and proteasomal degradation, resulting in abrogation of NF-κB activation [26]–[28]. Moreover, SOCS1 proteins are also known to regulate TLR-induced NF-κB activation by direct interaction with p65 subunit of NF-κB, causing downregulation of p65 protein levels by ubiquitin-mediated degradation [29].

Although MFG-E8 is a potential therapeutic agent in terms of downregulating the expression of pro-inflammatory cytokines in various inflammatory conditions [5], [30]–[32], the mechanisms underlying these features were not clearly elucidated. Herein, we aimed to evaluate the direct anti-inflammatory effects of MFG-E8 on LPS-induced TNF-α production in macrophages and focused on its plausible mechanism by which MFG-E8 transduce down-stream signaling for attenuating inflammation.

## Materials and Methods

### Experimental animals

Male weight (25–30 g) and age-matched wild-type (WT) C57BL/6J mice purchased from Taconic, Albany, NY and MFG-E8^−/−^ mice obtained as a kind gift from Dr. Shigekazu Nagata, Kyoto University, Japan, were housed in a temperature-controlled room on a 12 h light-to-dark cycle and fed a standard laboratory diet. Animal experimentation was carried out in accordance with the Guide for the Care and Use of Laboratory Animals (Institute of Laboratory Animal Resources). The project was approved by the Institutional Animal Care and Use Committee (IACUC) of The Feinstein Institute for Medical Research.

### Mouse peritoneal macrophage isolation and culture

The mice were euthanized by isoflurane inhalation, and then thioglycollate-elicited peritoneal exudates were collected by washing with ice-cold Ca^2+^ and Mg^2+^-free Hank's Balanced Salt Solution (HBSS), with 10% FBS and 100 units/mL each of penicillin (Invitrogen, CA) and streptomycin (Invitrogen). Collected peritoneal fluids were centrifuged at 400 *g* for 10 minutes (min) at 4°C and the resulting pellet was dissolved in 200 µl of red blood cell (RBC) lysis reagent for 2–3 min at 37°C. After centrifugation and washing with cold PBS, the RBC free pellet was dissolved in culture medium consisting of RPMI 1640 (Invitrogen) supplemented with 25 mM HEPES, 2 mM glutamine, 10% fetal bovine serum (FBS; Solon, Ohio), penicillin (100 IU/ml), and streptomycin (100 IU/ml). Peritoneal macrophages were then allowed to adhere in 24-well microplates at a density of 1×10^6^ cells/mL for 2 h at 37°C in a 5% CO_2_. Non-adherent cells were removed by washing with pre-warmed culture medium, and used in subsequent *in vitro* studies.

### Mouse model of endotoxemia

WT and MFG-E8^−/−^ mice were intraperitoneally (i.p.) injected with LPS (*Escherichia coli* O55:B5, Difco Laboratories, Detroit, MI) at a dose of 5 mg/kg BW for 4 h, and then blood was drawn from the inferior vena-cava. Serum was separated from whole blood by centrifugation at 4000 *g* and used for TNF-α ELISA. Mice injected with equal volume of saline instead of LPS served as sham control. To assess splenic TNF-α content, LPS (5 mg/kg BW) was i.p. injected into WT and MFG-E8^−/−^ mice, and then they were euthanized and the spleen harvested. Total RNA was extracted from 10 mg of splenic tissues, following reverse transcription, TNF-α mRNA expression was measured by real-time PCR using specific primers. Protein levels of TNF-α from the splenic tissue samples were examined by using ELISA from the whole tissue lysates.

### Cell line and culture condition

Mouse macrophage cell line, RAW264.7 cells obtained from American Type Culture Collection (ATCC), were cultured in DMEM media (Invitrogen) with 10% FBS and penicillin and streptomycin and kept in 37°C incubator under humidified condition containing 5% CO_2_.

### In vivo and in vitro treatment of recombinant murine MFG-E8 (rmMFG-E8)

WT mice were injected with rmMFG-E8 (R&D Systems, Minneapolis) at a dose of 0.4 µg/20 g BW via i.p. for 2 h. The subsequent studies were carried-out by initiating endotoxemia in mice with an overdose of LPS (5 mg/kg BW) injection into the peritoneal cavity. For *in vitro* experiments, peritoneal macrophages and RAW264.7 cells were pre-treated with 500 ng/mL rmMFG-E8 for 2 h, followed by LPS (10 ng/mL) stimulation, and used in several experiments.

### Quantitative real-time PCR

Total RNA extracted from mouse peritoneal macrophages and RAW264.7 cells by using the Trizol reagent (Invitrogen) was reverse-transcribed into cDNA. TNF-α and β-actin real-time PCR was performed using 7300 Real-Time PCR system (Applied Biosystems) with the primer sequences for TNF-α (NM_013693.2) forward: 5′-AGA CCC TCA CAC TCA GAT CAT CTT C-3′, reverse: 5′-TTG CTA CGA CGT GGG CTA CA-3′; and β-actin (NM_007393.3) forward: 5′-CGT GAA AAG ATG ACC CAG ATCA-3′, reverse: 5′-TGG TAC GAC CAG AGG CAT ACA G-3′. The reaction was carried out in a 25 µl mixture containing 0.1 µmol of each forward and reverse primers, 2 µg cDNA and 12.5 µl SYBR Green PCR Master Mix (Roche Diagnostics, Indianapolis, IN). The gene expression was normalized with that of β-actin as internal control and expressed as relative change to untreated samples.

### Enzyme-linked immunosorbent assay (ELISA)

TNF-α and IL-1β from the mouse macrophage cell culture supernatants pre-treated with and without rmMFG-E8 and treated with LPS were measured by ELISA using manufacturer's instructions (BD Biosciences, CA).

### Western blot analysis

Protein extraction and Western-blot assays were performed as described previously [30]. Briefly, RAW264.7 cells and spleen tissue samples were completely lysed in 10 mM Tris-HCl, pH 7.6, containing 2 mM Na orthovanadate pH 7.6, 0.2 mM PMSF, 2 µg/ml leupeptin, 2 µg/ml aprotinin and 1 tablet of Complete Mini (Roche Diagnostic, IN, USA). Equal amount of protein per lane was loaded into the 4–12% Bis-Tris gel (Invitrogen) and transferred to a 0.2-µm nitrocellulose membrane (Invitrogen). The membrane was incubated for overnight at 4°C with the primary antibodies (Ab) as obtained from respective vendors: rabbit anti-mouse pSTAT3 monoclonal Ab, and rabbit anti-mouse SOCS3 monoclonal Ab from Cell Signaling Technology, Danvers, MA and rabbit anti-mouse NF-κB (p65) from Santa Cruz Biotechnology, CA at a 1∶1000 dilution, reacted with peroxidase-conjugated goat anti-rabbit secondary Ab (Southern Biotech, AL) at a 1∶10,000 dilution at RT for 2 h, and washed five times in TBST. The resulting signals were detected by ECL (GE Healthcare, Buckinghamshire, UK). The immunoblot was washed and reprobed with mouse anti-β-actin Ab (Sigma-Aldrich, St. Louis, MO) as an internal control.

### Immunoprecipitation assay

Immunoprecipitation was performed as described previously [31]. Briefly; RAW264.7 cells were lysed in buffer containing 150 mM NaCl, 1% Nonidet P-40, 50 mM Tris HCl (pH 8.0), and 1 mM PMSF; and a total of 80 µg protein was pre-cleared for 1 h at 4°C with protein G-Sepharose beads (GE Healthcare). Pre-cleared extracts were immunoprecipitated overnight at 4°C with anti-mouse SOCS3 Ab and rabbit anti-mouse NF-κB (p65), followed by 1 h incubation with 50% slurry of protein G beads at 4°C in a mild rotating shaker, the beads were recovered by spinning at 10,000 *g* for 1 min. After 5 times washing, the beads were re-suspended in 30 µl of sample buffer and were subjected to Western blotting using anti-p65 Ab and rabbit anti-mouse Ubiquitin Ab (Cell Signaling Technology).

### Preparation of cytoplasmic and nuclear extract

Cytoplasmic and nuclear extracts were prepared as described previously [33]. RAW264.7 cells in 10-cm dishes were treated with rmMFG-E8 for 2 h, and then stimulated with LPS for 30 min. Cells were then washed two times with ice-cold phosphate-buffered saline (PBS) and resuspended in 250 µl of homogenization buffer containing 10 mM HEPES at pH 7.2, 3 mM MgCl_2_, 10 mM KCl, 3 mM NaCl, 1 mM EDTA, 1 mM EGTA (pH 7.5), 0.5 mM PMSF, 1 µg/ml leupeptin, 1 µg/ml aprotinin, and 2 mM Na orthovanadate (pH 7.6). The cells were allowed to swell on ice for 15 min, lysed gently with 12.5 µl of 10% NP-40, and centrifuged at 10,000 *g* for 10 min at 4°C. The supernatant was collected and used as the cytoplasmic extracts. The nuclear pellet was resuspended in 60 µl of nuclear extraction buffer containing 20 mM HEPES, pH 7.2, 1 mM MgCl_2_, 800 mM KCl, 25% glycerol, 0.5 mM EDTA, 1% NP-40, 0.5 mM PMSF, 1 µg/ml leupeptin, 1 µg/ml aprotinin, 2 mM Na orthovanadate (pH 7.6), agitated for 30 min at 4°C, and the nuclear debris was centrifuged at 10,000 *g* for 15 min. The supernatant (nuclear extract) was collected, frozen in liquid nitrogen and stored at −80°C until ready for analysis. Protein concentration was determined using Bradford reagent. Both the cytoplasmic and nuclear proteins were subjected to Western blotting using anti-p65 Ab, and reprobed with anti-GAPDH Ab (Santa Cruz Biotechnology) and anti-Histone Ab (Cell Signaling Technologies), respectively.

### Statistical analysis

Data are expressed as means ± SE and compared by one-way ANOVA and Student-Newman-Keuls (SNK) test. Student *t* test was used when only two groups were compared. Differences in values were considered significant if p<0.05.

## Results

### Pre-treatment of rmMFG-E8 attenuates LPS-induced TNF-α production in macrophages

**Figure 1:**
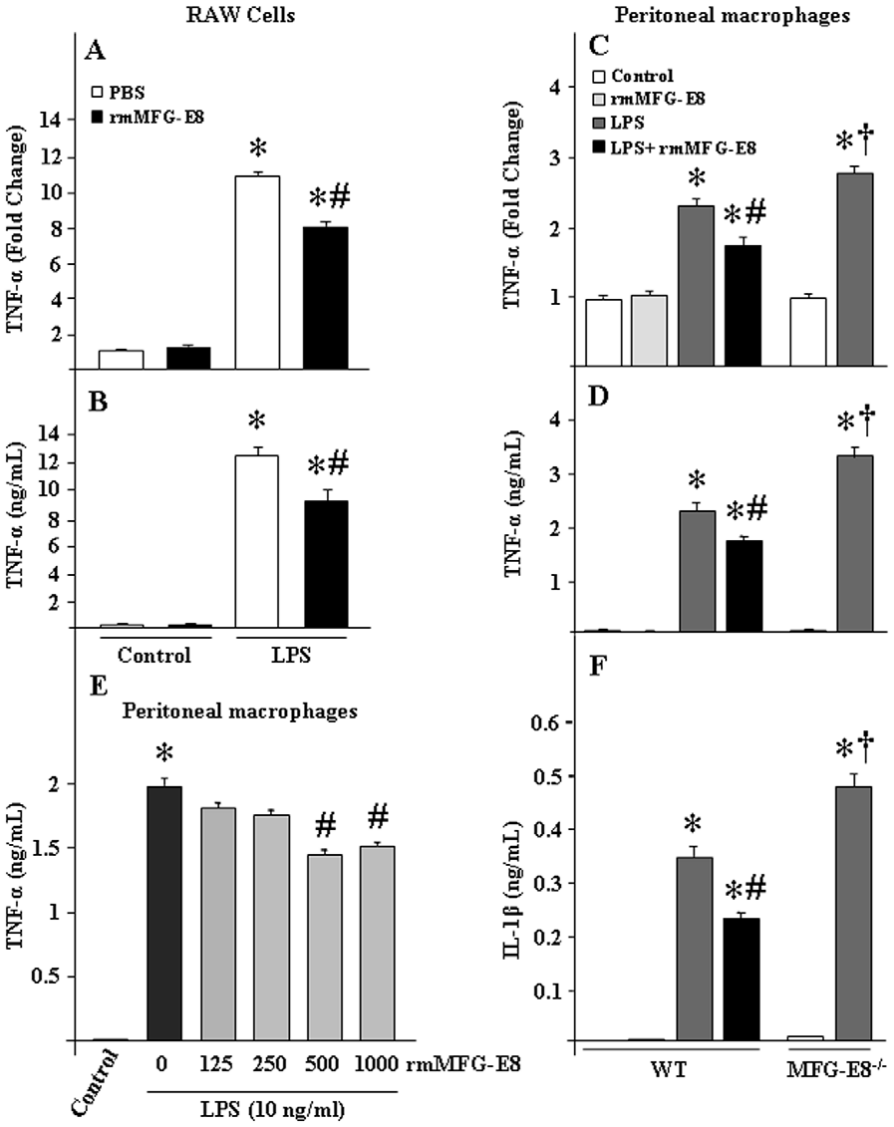
Effect of rmMFG-E8 on TNF-α production by LPS treated macrophages. (A, B) RAW264.7 cells (1×10^6^ cells) and (C, D) peritoneal macrophages from WT and MFG-E8^−/−^ mice (0.5×10^6^ cells) were plated in 24-well cell culture plates. After pre-incubation with rmMFG-E8 (500 ng/mL) for 2 h, the cells were then stimulated by LPS (10 ng/mL) for 2 h and the expression of TNF-α mRNA (A, C) was checked by real-time PCR. To assess the protein levels of TNF-α (B, D), RAW264.7 cells and mouse peritoneal macrophages were stimulated with LPS for 4 and 24 h, respectively and then ELISA was performed from the culture supernatants. (E) Dose-dependent effects of rmMFG-E8 for inhibiting the LPS-induced TNF-α production in murine peritoneal macrophages (0.5×10^6^ cells) cultured in 24-well cell culture plates. (F) Peritoneal macrophages from WT and MFG-E8^−/−^ mice (0.5×10^6^ cells) were plated in 24-well cell culture plates. After pre-incubation with rmMFG-E8 (500 ng/mL) for 2 h, the cells were stimulated by LPS for 24 h, followed by the measurement of IL-1β by ELISA. Data are expressed as means ± SE (n = 3 independent experiments) and compared by one-way ANOVA and SNK method: *P<0.05 vs. control; ^#^ *P*<0.05 vs. LPS and compared to rmMFG-E8; ^†^ *P*<0.05 vs. LPS and compared to MFG-E8^−/−^.

To determine the beneficial effects of MFG-E8, RAW264.7 cells as well as mouse peritoneal macrophages were incubated with rmMFG-E8, followed by the stimulation with LPS and TNF-α levels were assessed. As shown in Fig. 1A and 1B, RAW264.7 cells treated with LPS dramatically induced the TNF-α, while pre-treatment of rmMFG-E8 significantly reduced the LPS-induced TNF-α mRNA and protein levels by 25% and 29%, respectively. Similar effects were also observed in peritoneal macrophages from WT mice, where the cells treated with rmMFG-E8 significantly downregulated the LPS-induced TNF-α mRNA and protein levels by 24% and 23%, respectively (Fig. 1C, 1D).

MFG-E8 is abundantly expressed in macrophages. To reveal the role of endogenous MFG-E8, peritoneal macrophages isolated from MFG-E8^−/−^ mice were treated with LPS and then TNF-α production was assessed. We noticed that in LPS-treated conditions TNF-α mRNA and protein in peritoneal macrophages isolated from the MFG-E8^−/−^ mice were found to be significantly higher by 28% and 42% than those in WT, respectively (Fig. 1C, 1D).

Previously, several *in vitro* studies on immune reactive cells have been performed in an optimized dose of 500 ng/ml of rmMFG-E8 [31], [34]. Although supportive evidences exist, we carried out a pilot study where the dose dependent effects of rmMFG-E8 was evaluated in the context of downregulation of LPS-induced TNF-α production in murine peritoneal macrophages. Our results showed that the macrophage cells treated with 1000 and 500 ng/ml of rmMFG-E8 were able to exhibit significant downregulation of LPS-induced TNF-α production. On the other hand, no such significant effects were observed at lower doses of rmMFG-E8 (250 and 125 ng/ml) (Fig. 1E). We therefore, performed our *in vitro* studies in macrophages with 500 ng/ml of rmMFG-E8. Conversely, to attain the maximum response of rmMFG-E8 for downregulating the LPS-induced TNF-α production in macrophages, we optimized the LPS concentration by stimulating the murine macrophage cells at various concentrations of LPS while keeping the rmMFG-E8 pre-treatment dose and incubation time constant. We noticed that the rmMFG-E8-mediated significant reduction of TNF-α levels in macrophages, was obtained at 10 ng/ml of LPS stimulation but not in lower doses (<10 ng/ml) (data not shown). Hence we selected the 10 ng/ml of LPS dose for subsequent *in vitro* studies.

Besides TNF-α, the roles of other pro- and anti-inflammatory cytokines and chemokines are also critical. While doing the *in vitro* experiments, we noticed that the RAW264.7 cells did not produce IL-1β, IL-6 and IL-10 if treated with LPS alone, but rather required stimulation with LPS in combination with interferon-γ (IFN-γ). We therefore, using the murine peritoneal macrophages evaluated the effects of rmMFG-E8 in terms of downregulating the production of pro-inflammatory cytokine, other than TNF-α. According to our results, we found that the pre-treatment of rmMFG-E8 significantly downregulated the IL-1β levels (34.2%) in LPS treated WT murine peritoneal macrophages (Fig. 1F). In contrast, the IL-1β contents were significantly higher (37%) in LPS-treated peritoneal macrophages isolated from MFG-E8^−/−^ mice as compared to WT animals (Fig. 1F). Taken together, these results demonstrated the novel anti-inflammatory roles of MFG-E8 as generated not only by downregulating the TNF-α but also by reducing IL-1β levels in macrophages.

### Mice pre-treated with rmMFG-E8 were protective against LPS-induced endotoxemia by reducing TNF-α

**Figure 2:**
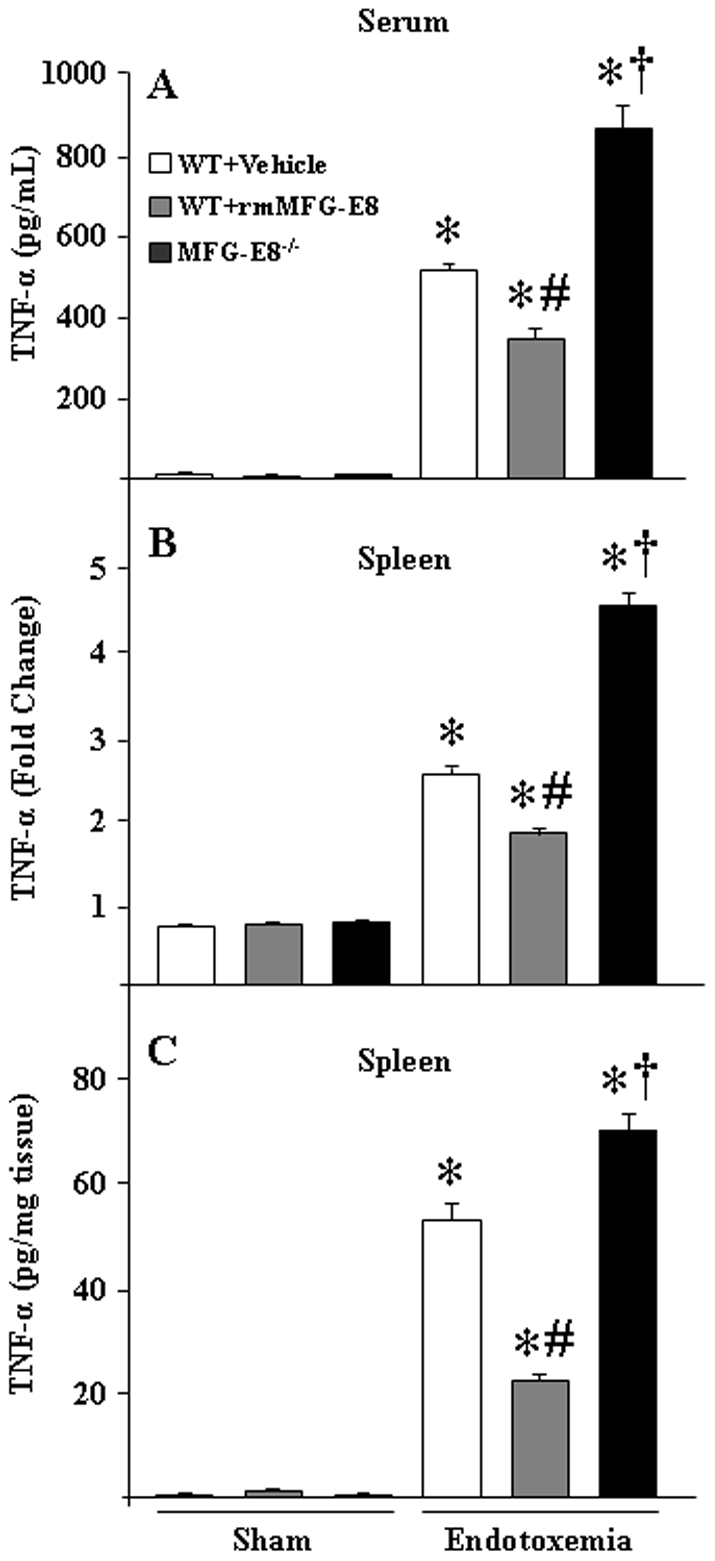
In vivo effects of rmMFG-E8 in controlling TNF-α production during endotoxemia. (A) WT mice were injected with rmMFG-E8 (0.4 µg/20 g BW) via i.p. for 2 h and then endotoxemia was generated by an overdose of i.p LPS (5 mg/kg BW) administration. Similarly, in another experiment, WT and MFG-E8^−/−^ mice were treated with LPS (5 mg/kg BW) via i.p.. After 4 h, mice were euthanized, blood was collected and TNF-α measured by ELISA. (B, C) WT mice were treated with rmMFG-E8 (0.4 µg/20 g BW) for 2 h, followed by LPS (5 mg/kg BW) challenge. WT and MFG-E8^−/−^ mice were injected with LPS (5 mg/kg BW) i.p.. After 2 h total RNA and after 4 h proteins were extracted from splenic tissues for TNF-α expression using real-time PCR and ELISA, respectively. All data are expressed as means ± SE (n = 3 independent experiments) and compared by one-way ANOVA and SNK method: *P<0.05 vs. sham control; ^#^ *P*<0.05 vs. endotoxemia and compared to rMFG-E8 treatment; ^†^ *P*<0.05 vs. endotoxemia and compared to MFG-E8^−/−^.

We have previously demonstrated that during sepsis or in LPS-treated conditions, endogenous MFG-E8 expression in the spleen, macrophages and in circulation was dramatically reduced [34], [35]. To compensate the deficit of MFG-E8 that occurred during LPS-treatment, we administered rmMFG-E8 exogenously into the WT mice and examined its effects in TNF-α production during endotoxemia. As shown in Fig. 2A, WT mice injected with rmMFG-E8 showed a significant decrease in serum levels of TNF-α after *in vivo* LPS administration as compared to vehicle treated mice. Conversely, the i.p. LPS injection markedly increased the serum contents of TNF-α in MFG-E8^−/−^ mice as compared to the WT counterparts (Fig. 2A); hence showing concordance with our *in vitro* results using peritoneal macrophages from these two groups of mice (Fig. 1C, 1D).

MFG-E8 serves as an essential factor for the phagocytic engulfment of apoptotic cells by macrophages. Studies with MFG-E8^−/−^ mice showed infiltrating apoptotic cells at the germinal centers of spleen [2], [3]. Considering that the spleen as one of the vital organs in innate immune system, we noticed an enlarged size and weight of the spleen in MFG-E8^−/−^ mice as compared to the WT mice (data not shown), pointing to the possibility of an abnormal immune function in the spleen of those mice groups. Hence, we intended to compare the splenic contents of TNF-α in endotoxemic mice treated with rmMFG-E8. Our results showed that the mRNA and protein levels of TNF-α in rmMFG-E8 treated WT mice were significantly reduced as compared to non-treated mice with endotoxemia (Fig. 2B, 2C). Furthermore, although significant induction of TNF-α levels in both WT and MFG-E8^−/−^ mice in the spleen was observed after i.p. endotoxin challenge, the levels were comparatively higher in MFG-E8^−/−^ mice than those in WT mice (Fig. 2B, 2C). Collectively, these evidences strongly supported the notion of MFG-E8-mediated downregulation of TNF-α production in endotoxemia.

### STAT3 is activated in macrophages after rmMFG-E8 pre-treatment

**Figure 3:**
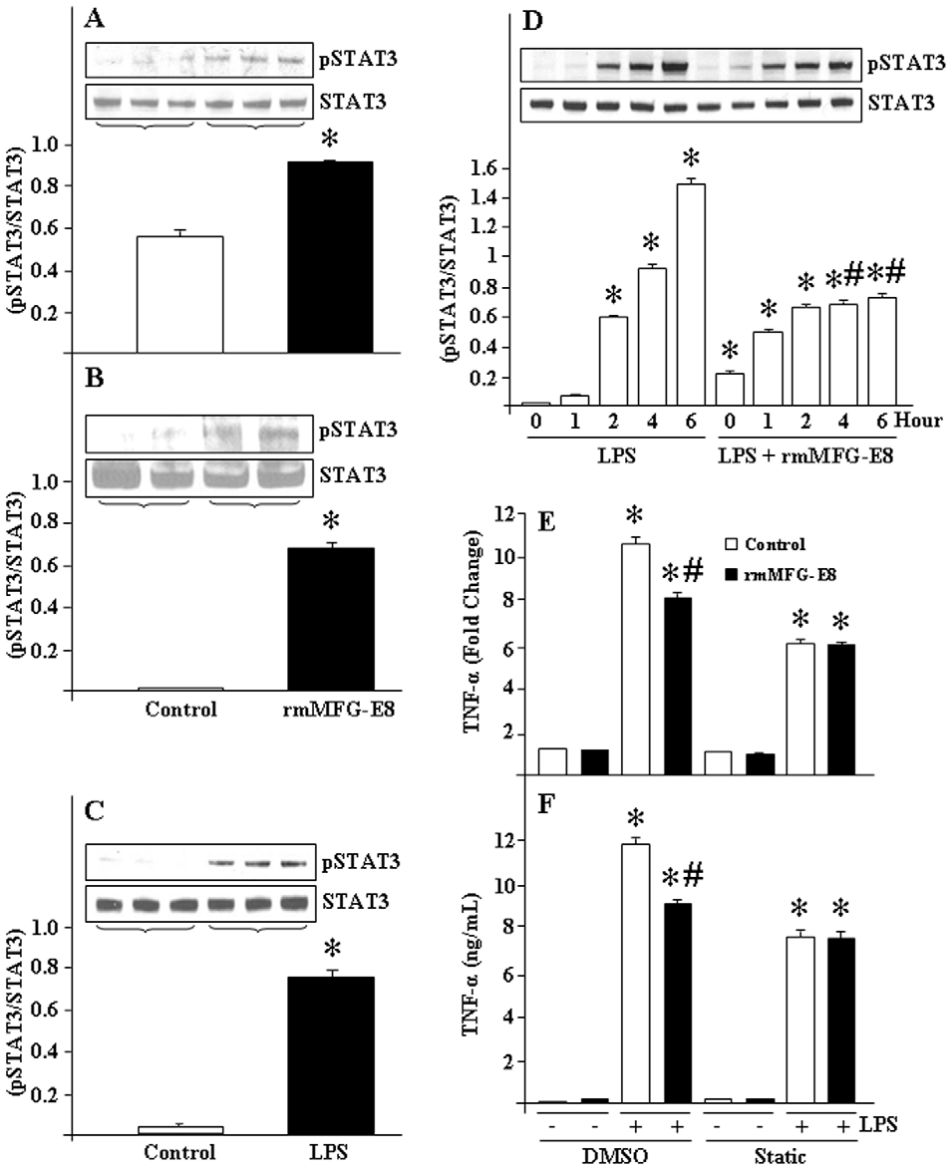
MFG-E8 activates pSTAT3. Western-blot for pSTAT3 was done in extracted total proteins from (A) RAW264.7 cells (4×10^6^ cells) and (B) mouse peritoneal macrophages (2×10^6^ cells) grown in 6-well plate and treated with rmMFG-E8 (500 ng/mL) for 2 h. (C) RAW264.7 cells were cultured and then stimulated with LPS (10 ng/mL) for 2 h. Western-blot analysis was performed from 25 µg of extracted proteins from the above described experiments using anti-mouse pSTAT3 Ab. The immunoblots were stripped and reprobed with anti-mouse STAT3 Ab. The images shown are representatives of 3 independent experiments. Densitometric evaluations (pSTAT3/STAT3) are expressed as means ± SE and compared by one-way ANOVA and SNK method: *P<0.05 vs. control. (D) Total protein was extracted from RAW264.7 cells (1×10^6^ cells) grown in 24-well culture plates and treated with rmMFG-E8 (500 ng/mL) and LPS (10 ng/mL) at different time points, and Western-blot for pSTAT3 and STAT3 was performed as described above. A representative autoradiograph is also shown. Densitometric evaluations (pSTAT3/STAT3) are expressed as means (n = 3) ± SE and compared by one-way ANOVA and SNK method: *P<0.05 vs. LPS at 0 h; ^#^ *P*<0.05 vs. LPS at corresponding time points. (E, F) RAW264.7 cells (1×10^6^ cells) were cultured in a 24-well cell culture plate and treated with Static (6.25 µM) for overnight, followed by pre-treatment with rmMFG-E8 (500 ng/mL) for 2 h and stimulated with LPS (10 ng/mL) for another 4 h. TNF-α mRNA and protein were measured by real-time PCR and ELISA, respectively. For real-time PCR, β-actin served as the internal control. Data are expressed as means (n = 3) ± SE and compared by one-way ANOVA and SNK method: *P<0.05 vs. LPS (−); ^#^ *P*<0.05 vs. LPS (+).

To address the mechanisms by which MFG-E8 functions to downregulate the LPS-induced TNF-α production in macrophages, we performed *in vitro* experiments using RAW264.7 cells and mouse peritoneal macrophages. Within 2 h of rmMFG-E8 treatment, the phosphorylation of STAT3 (pSTAT3) was significantly induced in RAW264.7 cells (Fig. 3A), and in mouse peritoneal macrophages (Fig. 3B). However, LPS can also robustly induce the pSTAT3 levels in 2 h, which may further implicate it as a potent transcription factor for increasing TLR4-mediated TNF-α production (Fig. 3C). Next, we attempt to examine the status of STAT3 in conditions where the cells were pre-treated with rmMFG-E8, followed by exposure with LPS. As shown in Fig. 3D, using RAW264.7 cells, we noticed that LPS-induced STAT3 activation started from 2 h and commenced up to prolonged time course of at least 6 h. An interesting feature as evident from our study revealed that when the cells were pre-treated with rmMFG-E8, the overall LPS effects for STAT3 activation become reduced at least in part in a time course dependent manner (Fig. 3D). This could be due to the fact that the pre-treatment of rmMFG-E8 may induce the expression of down-stream negative regulator(s) to interfere with the LPS-induced STAT3 signaling.

To verify the STAT3 roles as a down-stream mediator for LPS-induced optimal TNF-α production, we used a chemical inhibitor of STAT3, Static, to treat RAW264.7 cells and then LPS effects for TNF-α expression were assessed. With an optimized dose of Static at which it inhibits STAT3 phosphorylation without affecting the cell viability, we noticed that the effects of LPS on TNF-α mRNA and protein were decreased in Static-treated cells as compared to the DMSO-treated controls (Fig. 3E, 3F), suggesting the requirement of STAT3 for LPS-induced optimal TNF-α production in macrophages. Furthermore, due to the blocking of STAT3 functions by Static, we noticed that the beneficial effects of MFG-E8 for downregulating the LPS-induced TNF-α production was diminished, suggesting the pivotal role of STAT3 for MFG-E8-mediated downregulation of TNF-α contents (Fig. 3E, 3F). Therefore, STAT3 may provide dual roles: one as the LPS induced pro-inflammatory factor, while the other as the MFG-E8- mediated anti-inflammatory signal.

### MFG-E8 pre-treatment upregulates SOCS3 expression in macrophages

**Figure 4:**
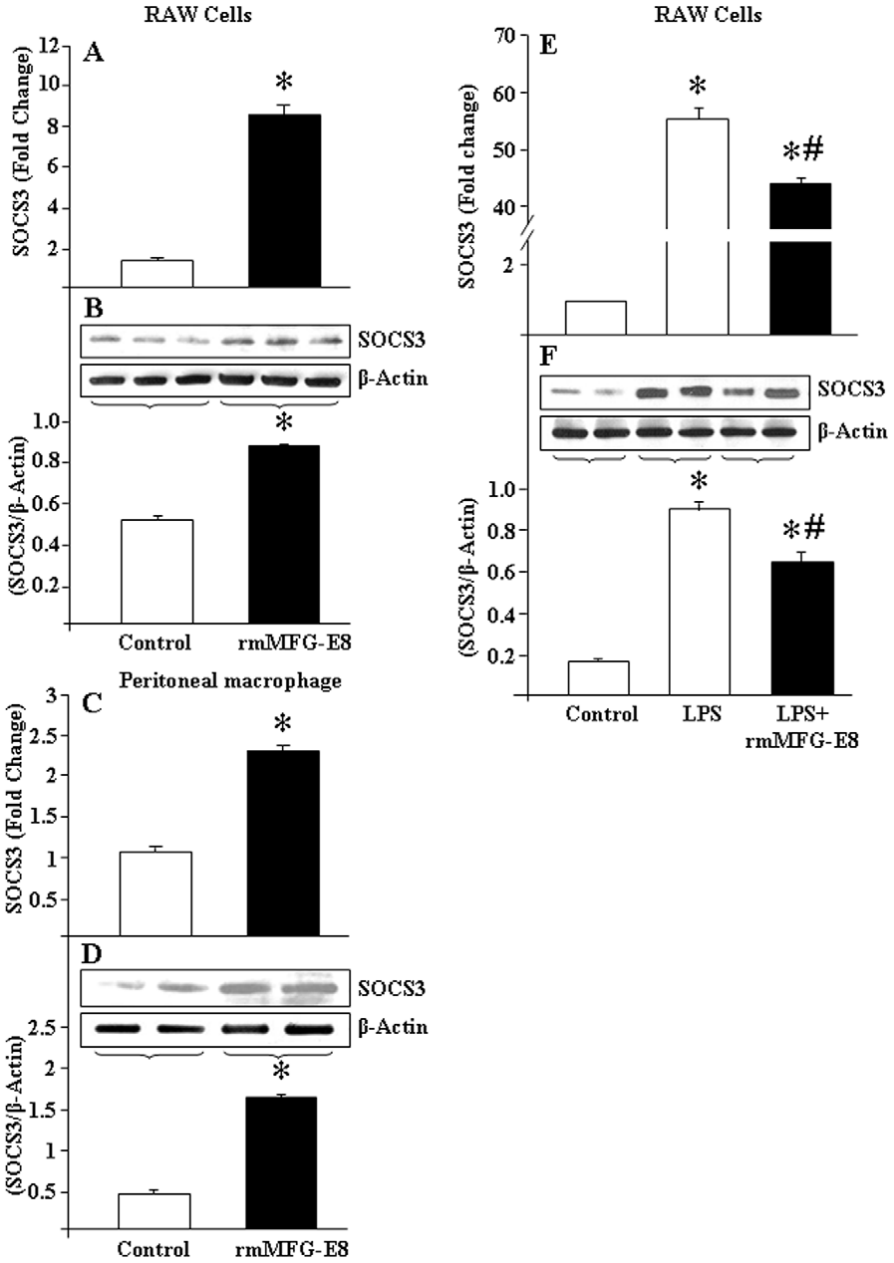
MFG-E8 induces SOCS3 expression in macrophages. (A, B) RAW264.7 cells (4×10^6^ cells) and (C, D) mouse peritoneal macrophages (2×10^6^ cells) were cultured in a 6-well cell culture plates, and treated with rmMFG-E8 (500 ng/mL) for 2 h followed by assessment of SOCS3 mRNA expression and protein using real-time PCR and Western blot analysis, respectively. For real-time PCR, β-actin served as the internal control. To examine protein level of SOCS3, a total of 25 µg of proteins extracted from each samples were subjected for Western-blotting. The image shown is representative of results obtained from 3 independent experiments. The immunoblot was reprobed with mouse anti-β-actin Ab as loading control. Densitometric evaluations (SOCS3/β-actin) are expressed as means ± SE and compared by one-way ANOVA and SNK method: *P<0.05 vs. control. (E, F) RAW264.7 cells (4×10^6^ cells) were cultured in a 6-well cell culture plate in presence of rmMFG-E8 (500 ng/mL) for 2 h, followed by LPS (10 ng/mL) stimulation for 2 and 4 h, respectively, for SOCS3 mRNA expression by real-time PCR and protein levels by Western-blotting. In each experiment, β-actin served as the internal control. Results are expressed as means ± SE and compared by one-way ANOVA and SNK method. *P<0.05 vs. control; ^#^ *P*<0.05 vs. LPS.

To reveal whether MFG-E8 plays any role in activating the down-stream negative regulator, RAW264.7 cells were treated with rmMFG-E8. The result showed significant induction of SOCS3 expression at the mRNA and protein levels (Fig. 4A, 4B). Similar results were also observed in murine peritoneal macrophages (Fig. 4C, 4D). During LPS treatment, RAW264.7 cells dramatically induced the SOCS3 expression at its transcriptional and translational levels, while the effects of LPS for SOCS3 become significantly decreased if the cells were pre-treated with rmMFG-E8 (Fig. 4E, 4F). This would suggest the possibility of negative regulation of TLR4 cascades mediated by SOCS3 that was upregulated during the 2 h rmMFG-E8 pre-treatment.

### In vivo LPS exposure augments STAT3 and SOCS3 in mouse peritoneal cells and splenic tissues

**Figure 5:**
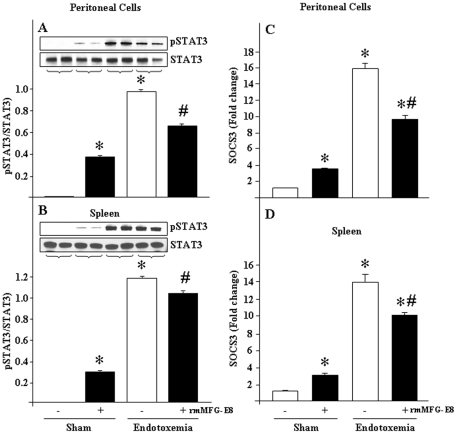
In vivo effects of rmMFG-E8 on pSTAT3 and SOCS3 expression during endotoxemia. WT mice were injected with rmMFG-E8 (0.4 µg/20 g BW) via i.p. for 2 h and then endotoxemia was generated by an overdose of LPS (5 mg/kg BW) administration via i.p route. After 2 h of LPS treatment mice were euthanized and total proteins were extracted from (A) peritoneal cells and (B) spleen to perform Western-blot using anti-pSTAT3/STAT3 Abs. On the other hand, SOCS3 expression was assessed by real-time PCR using each of the cDNAs extracted from above samples of (C) peritoneal cells and (D) spleen. β-actin served as an internal control. Data are expressed as means ± SE and compared by one-way ANOVA and SNK test from 3 independent experimental results. *P<0.05 vs. sham control; ^#^ *P*<0.05 vs. endotoxemia.

To evaluate the *in vivo* status of STAT3 and SOCS3 levels in endotoxemic mice after pre-treatment with rmMFG-E8, we carried out Western blot for phospho and total STAT3 levels and real-time PCR for SOCS3 expression from collected peritoneal cells and splenic tissues. After 2 h of rmMFG-E8 pre-treatment, pSTAT3 become significantly increased in peritoneal cells and splenic tissues (Fig. 5A, 5B). Moreover, we also noticed that the mice treated with LPS showed dramatic induction of pSTAT3 levels in those samples, while that was comparatively lower in endotoxemic mice pre-treated with rmMFG-E8 *in vivo* (Fig. 5A, 5B). Consistent with the above findings, we also obtained similar trend in results for SOCS3 gene expression in splenic tissue samples. When the mice were given rmMFG-E8 i.p., the SOCS3 expression in peritoneal cells and spleen was significantly induced, which become further elevated during endotoxemia by i.p. LPS injection, but comparatively decreased in levels if they were pre-treated with rmMFG-E8 followed by endotoxemia (Fig. 5C, 5D). Since SOCS3 is a target gene of STAT3, hence modulation of STAT3 phosphorylation during LPS and rmMFG-E8 pre-treatment may in turn influence the SOCS3 expression under these conditions.

### SOCS3 is activated via STAT3 pathway and targets NF-κB p65 during rmMFG-E8 pre-treatment

**Figure 6:**
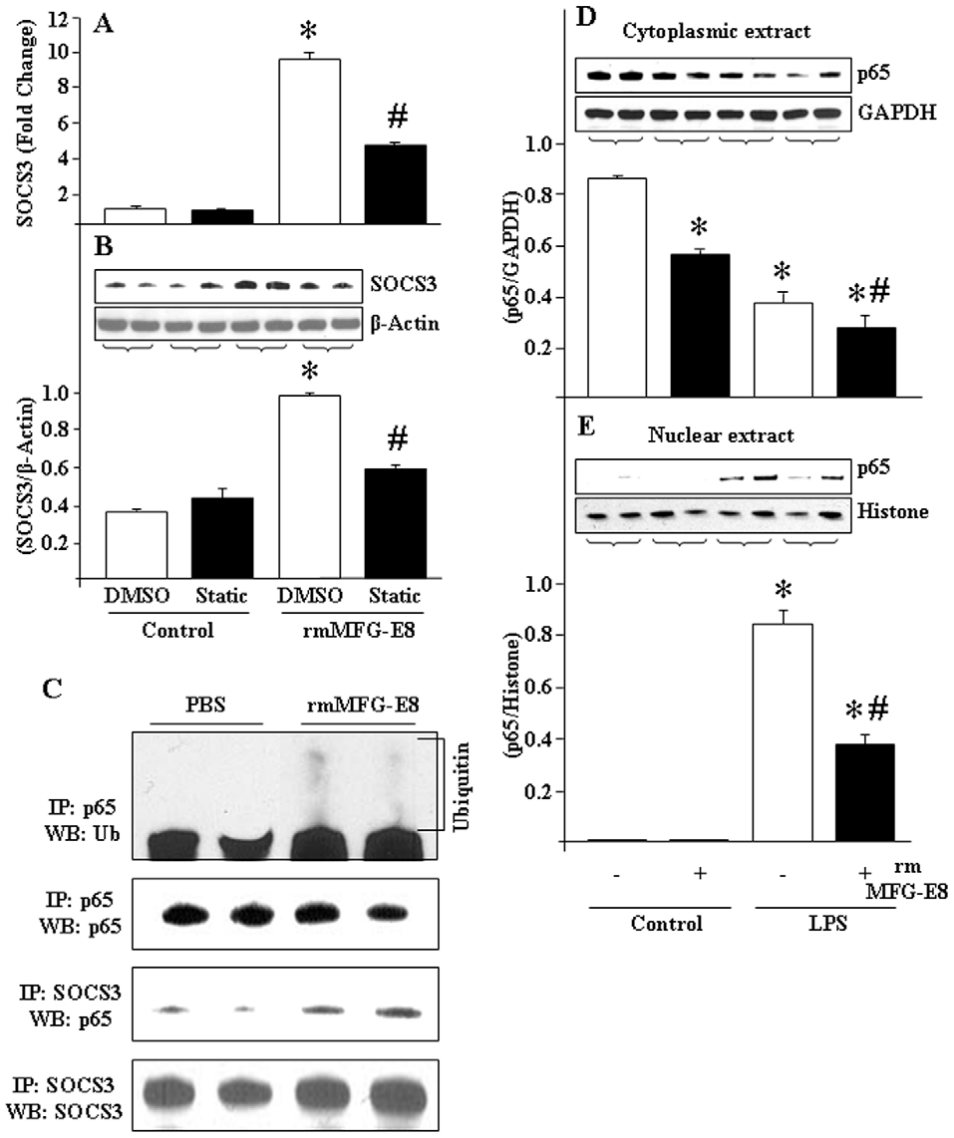
Interaction between NF-κB p65 and SOCS3 in rmMFG-E8-treated RAW264.7 cells. (A, B) RAW264.7 cells (4×10^6^ cells) were grown into the 6-well culture plates and incubated for overnight in presence of Static (6.25 µM), followed by rmMFG-E8 (500 ng/mL) treatment for 2 h. Real-time PCR was done for SOCS3 mRNA expression (A) and Western blotting for SOCS3 protein levels (B). In each samples, the expression of β-actin served as an internal control. (B) The protein level of SOCS3 was assessed by Western-blot from equal amounts (25 µg) of proteins and the image shown is representative of 3 independent experiments. The immunoblot was reprobed with mouse anti-β-actin Ab as loading control. Densitometric evaluations (SOCS3/β-actin) are expressed as means ± SE and compared by one-way ANOVA and SNK method: *P<0.05 vs. DMSO Control; ^#^ *P*<0.05 vs. DMSO+rmMFG-E8. (C) RAW264.7 cells (4×10^6^ cells) were cultured in a 6-well cell culture plates, and treated with rmMFG-E8 (500 ng/mL) for 2 h. Proteins (80–100 µg) were immunoprecipitated using anti-mouse SOCS3 Ab and anti-mouse p65 Ab then subjected to Western-blotting and immunoreacted with anti-p65 Ab for the detection of p65 protein and anti-ubiquitin Ab for detection of ubiquitination. The membrane was re-probed with mouse anti-SOCS3 and anti-p65 Ab for determining SOCS3 and p65 contents. (D, E) RAW264.7 cells (4×10^6^ cells) were grown in 6-well culture plates, and then pre-treated with rmMFG-E8 (500 ng/mL) for 2 h. After 30 min of LPS (10 ng/mL) stimulation (D) cytoplasmic and (E) nuclear proteins were extracted to perform Western-blot using anti-p65 Ab. The membranes were reprobed with anti-GAPDH and Histone Abs for evaluating the loading status of cytoplasmic and nuclear proteins, respectively. The images shown are representatives of 3 independent experiments. *P<0.05 vs. control; ^#^ *P*<0.05 vs. LPS.

Since the expression of SOCS3 is regulated by the upstream transcription factor STAT3 [16], we used Static to treat RAW264.7 cells and assessed rmMFG-E8 effects on SOCS3 expression to prove whether STAT3 is required for rmMFG-E8-dependent SOCS3 expression. With an optimized dose of Static, we noticed that the effects of rmMFG-E8 on SOCS3 activation was diminished at its mRNA and protein levels (Fig. 6A, 6B), confirming the requirement of STAT3 for MFG-E8-mediated SOCS3 expression in macrophages.

SOCS3 protein contains an extraordinary ubiquitin ligase activity for ubiquitination and proteosomal degradation of a number of intracellular signaling components at which it interacts [26], [29]. As such, we aimed to determine whether the SOCS3 as induced by rmMFG-E8 had any interaction with NF-κB p65 component. To attain this, RAW264.7 cells were treated with rmMFG-E8 for 2 h, and then the p65 contents coupled to SOCS3 protein was determined by immunoprecipitation from the total cellular extracts. As shown in Fig. 6C, RAW264.7 cells treated with rmMFG-E8 clearly showed an increased level of p65 protein immunoprecipitated with rmMFG-E8-induced SOCS3 protein. Since SOCS3 induces the ubiquitination of the target protein, we therefore assumed p65 protein might be ubiquitinated and degraded after rmMFG-E8 pre-treatment. To prove our speculation, proteins from the rmMFG-E8 pre-treated macrophages were subjected to immunoprecipitation by using anti-p65 Ab, followed by Western blotting using anti-ubiquitin Ab to assess ubiquitination. According to our findings, we clearly noticed the presence of ubiquitination in rmMFG-E8 pre-treated and p65 immunoprecipitated samples, while it was undetectable in control samples (Fig. 6C).

Since NF-κB activation for target gene transcription requires nuclear translocation of RelA/p65 subunit of NF-κB, we therefore examined the effect of rmMFG-E8 on the cytosolic and nuclear p65 protein levels in RAW264.7 cells treated with LPS. Herein, we obtained four different features of cytosolic vs. nuclear contents of p65 proteins depending on the combinations used with rmMFG-E8 and LPS (Fig. 6D, 6E). At the resting state of cells where neither rmMFG-E8 nor LPS was added, majority of the p65 protein was localized at the cytoplasmic fraction (Fig. 6D; lane 1, 2 from left), while undetected at the nuclear portion (Fig. 6E; lane 1, 2 from left). Cells treated with rmMFG-E8 showed decreased contents of cytoplasmic p65 (Fig. 6D; lane 3, 4 from left), instead of having any nuclear translocation of p65 (Fig. 6E; lane 3, 4 from left), suggesting the possibility of its proteolytic degradation during rmMFG-E8 treatment. In LPS-treated conditions, the cytoplasmic p65 was found to be decreased (Fig. 6D; lane 5, 6 from left), and correspondingly the nuclear contents were increased (Fig. 6E; lane 5, 6 from left), thus reflecting the nuclear translocation of p65 upon LPS treatment for target gene expression. On the other hand, if rmMFG-E8 pre-treated cells were stimulated by LPS, the cytoplasmic (Fig. 6D; lane 7, 8 from left), and the nuclear contents of p65 (Fig. 6E; lane 7, 8 from left) were comparatively lower than in cells treated with LPS alone (Fig. 6D and 6E; lane 5, 6 from left). Since rmMFG-E8 pre-treatment caused reduction of the cytoplasmic p65 contents, further LPS treatment in this condition might decrease the nuclear translocation of p65, resulting in decreased levels of target gene expression. Collectively, these evidences strongly support the notion of MFG-E8-mediated modulation of TLR4 signaling for attenuating inflammation.

## Discussion

Recently, MFG-E8 mediated therapeutic potential has been reported in several disease models. Although majority of the studies focused on the beneficial effects of MFG-E8 due to the phagocytic clearance of apoptotic cells, a few studies also suggested the role of MFG-E8 in innate-immune system regardless of its roles in clearance of the apoptotic cells from the body [6], [31], [32], [36]. Our study demonstrated the direct anti-inflammatory role of MFG-E8 in terms of attenuating TNF-α production in mouse peritoneal macrophages and RAW264.7 cells treated with LPS. Since TNF-α is one of the early responsive pro-inflammatory cytokines produced by macrophages during LPS treatment, the anti-inflammatory therapeutic agents are mainly based on neutralizing the excessive ectopic or systemic TNF-α release [37]. Although the deleterious effects of TNF-α have been reported immensely in various inflammatory conditions, the roles of other pro-inflammatory cytokines and chemokines, such as, interleukin-1β (IL-1β), IL-6, IL-12, IL-17 and IL-8 can not be ignored; hence the therapeutic potential may be attained by targeting these cytokines and chemokines. Using the murine peritoneal macrophages we evaluated the effects of rmMFG-E8 in terms of downregulating the production of other pro- and anti-inflamatory cytokines, IL-1β and IL-10 after treatment with LPS for 24 h. We found that LPS significantly induced the production of IL-1β, but not IL-10 in murine peritoneal macrophages at 24 h, and surprisingly the pre-treatment of rmMFG-E8 dramatically decreased the IL-1β levels, even higher than that of TNF-α in above conditions. Since, we could not detect IL-10 at 24 h of LPS stimulation in mouse peritoneal macrophages, we therefore, prolonged the incubation time up to 48 h, at which although the IL-10 levels was detectable but there was no additional increase with rmMFG-E8 pre-treatment (data not shown); hence the possibility of MFG-E8-mediated anti-inflammatory functions via IL-10 dependent STAT3 pathway may be refractory. In support of our current *in vitro* findings, several *in vivo* studies have also been conducted to prove the beneficial roles of exogenous rmMFG-E8 in sepsis, colitis, gut, renal and hepatic ischemia and reperfusion injuries by downregulating not only the TNF-α levels but also other pro-inflammatory cytokines in target tissues and serum samples [4], [5], [31].

In several *in vitro* and *in vivo* studies concerning the therapeutic potential, rmMFG-E8 protein was given as a pre-treatment regimen [31], [34]. In our current project, rmMFG-E8 was given as a pre-treatment regimen before LPS stimulation in macrophage cells or generating endotoxemia *in vivo*. However in some *in vivo* studies, instead of pre-treatment, rmMFG-E8 was given as post-treatment or at the same time of inducing the disease, and the beneficial roles of rmMFG-E8 in protecting the severity of the diseases was observed [4], [5]. Furthermore, in mouse model of colitis, rmMFG-E8 was injected intravenously before the onset of colitis and prolonged throughout the disease course as post treatment [31] However, in an *in vitro* system to reveal the indirect anti-inflammatory roles of rmMFG-E8 via accelerating the phagocytosis of apoptotic cells, rmMFG-E8 was added to the co-culture (apoptotic cells and phagocytes) as pre-treatment regimen and then the LPS-stimulated TNF-α production was measured [34]. From the above discussion, since the beneficial effects of rmMFG-E8 can be observed in any of the pre- and post-treatment condition, further studies using rmMFG-E8 as a post-treatment regimen towards delineating the anti-inflammatory signal transduction mechanisms might be of great interest.

TLRs are transmembrane proteins expressed by the cells of the innate immune system, which recognize the pathogen associated molecular patterns (PAMPs) and activate the signaling pathways that launch immune and inflammatory responses against invaders [38], [39]. In mammals, the TLR family includes eleven proteins (TLR1–TLR11). Different TLRs serve as receptors for diverse ligands, including bacterial cell wall components, viral double-stranded RNA and even small-molecule anti-viral or immunomodulatory compounds. In humans, TLR1, 2, 4, 5 and 6 are outer membrane associated, and respond primarily to bacterial surface associated PAMPs. The second group, TLR3, 7, 8 and 9 are found on the surface of endosomes, where they respond primarily to nucleic acid based PAMPs from viruses and bacteria. Upon binding with their cognates, TLRs activate the core pathway leading to activation of the transcription factor NF-κB and MAP kinases, e.g., p38 and JNK [9], [38], [39]. Abnormal functioning of TLR 2, 4, and 5 are noticed in sepsis, inflammatory bowel diseases (IBD), atherosclerosis, asthma, rheumatoid arthritis, Alzheimer's disease, while the activated TLR 7, 8, and 9 are seen in SLE, primary tumors, viral infection and skin lesions [40]. Targeting the TLRs by using the respective neutralizing antibodies, and small-molecule antagonists might also be prospects in disease therapy [40]. Since we studied the potential anti-inflammatory roles of MFG-E8 by utilizing the LPS induced murine model of endotoxemia, we therefore highlighted the features of TLR4-mediated innate immune responses upon rmMFG-E8 pre-treatment.

The anti-inflammatory effects of MFG-E8 have been reported in several studies [4], [5], [31]. In general, the phagocytic engulfment of apoptotic cells creates an immune-tolerant state at the site of inflammation by reducing the production of pro-inflammatory cytokines and enhancing anti-inflammatory cytokines [41]. Based on this concept, recent reports proposed an indirect mechanism where MFG-E8 accelerated the phagocytic clearance of apoptotic cells, thereby modulating the intra-cellular NF-κB, p38, ERK1/2 and JNK signaling for decreasing pro-inflammatory cytokine production [34]. Depending on this view, exogenous administration of rmMFG-E8 attenuated inflammation by reducing pro-inflammatory cytokines, TNF-α, IL-6 and IL-1β in sepsis as well as other diseases where LPS-TLR4 signaling is predominant [4], [5]. Interestingly, the high-mobility group box 1 protein (HMGB1) was reported to interfere with the normal functioning of MFG-E8 by competitively binding to the α_v_β_3_-integrin [42]. Similarly, one recent approach for MFG-E8-mediated anti-inflammatory role has been proposed, where MFG-E8 was found to abrogate the osteopontin (OPN)-mediated α_v_β_3_-integrin outside-in signaling, thereby inhibiting the synergistic effects for intensifying the inflammation [31]. Besides these, herein we proposed a novel mechanism for direct anti-inflammatory roles of MFG-E8 in terms of attenuating the LPS-induced TNF-α production in macrophages.

Treatment of murine macrophage cells by rmMFG-E8 significantly induced the STAT3 phosphorylation which in turn up-regulated the down-stream SOCS3 gene expression. By using the pSTAT3 inhibitor, we further proved that the induction of SOCS3 was mediated via STAT3 pathway. However, it would be interesting to elucidate how MFG-E8:α_v_β_3_-integrin signaling leads to STAT3 phosphorylation and SOCS3 upregulation in experimental systems. In viable cells, MFG-E8 signaling is mediated through the binding of its receptor, α_v_β_3_-integrin, expressed by variety of cells [1]. Integrin signaling is based on a complex network of intra-cellular components which may lead to cross talk among other pathways [43]. Once recognizing its ligand, α_v_β_3_-integrin signaling is transduced through a number of intracellular molecules and protein tyrosine kinases, e.g., caveolin, vincullin, talin, paxillin, focal adhesion kinase (FAK), Src, fyn and others [43]. In quest of finding any link between α_v_β_3_-integrin and STAT3, recent study has elucidated that OPN, one of the ligands of α_v_β_3_-integrin could activate the STAT3 signaling via binding to the α_v_β_3_-integrin for promoting the cell growth [44]. In another study, the roles of α_v_β_3_-integrin mediated Src tyrosine kinases for inducing the activation of STAT3 has been well demonstrated in intestinal epithelial cells, while the inhibition of Src prevented the activation of STAT3 [45]. From the above evidences, the MFG-E8-mediated STAT3 activation via α_v_β_3_-integrin pathway was anticipated.

**Figure 7:**
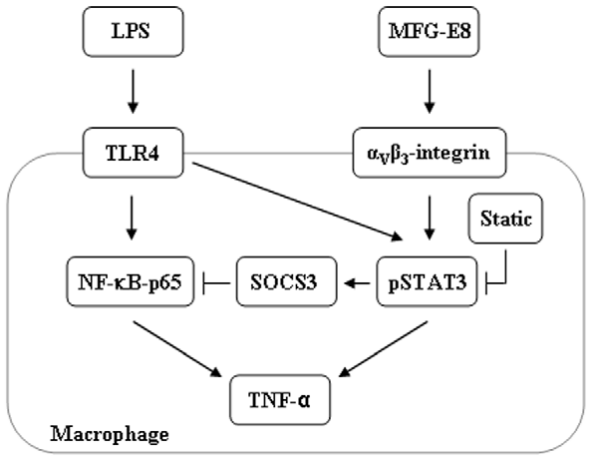
Hypothesis scheme. In macrophage cells, LPS triggers TLR4 pathway to activate NF-κB signals by promoting IκB degradation and nuclear translocation of p50 and RelA/p65 to enhance the target gene e.g., TNF-α transcription. In our current study, we demonstrated a non-canonical signaling where LPS may activate the phosphorylation of a ubiquitous transcription factor, STAT3 which in turn can further augment the down-stream signaling for TNF-α expression, since blocking STAT3 activation by Static dramatically reduced the LPS induced TNF-α production. In the biological system, the versatile function of STAT3 enabled it to generate both the inflammatory and anti-inflammatory signal transduction efficiently. Utilizing the STAT3 pathway, MFG-E8 can directly exert its anti-inflammatory effects towards downregulating the LPS-induced TNF-α production in macrophages via activating SOCS3. MFG-E8-induced SOCS3 may in turn target the NF-κB p65 component for ubiquitination and act as a negative regulator of LPS-mediated TLR4 signaling via NF-κB for TNF-α production.

During LPS stimulation, the cross-talk between TLR4 and STAT3 was observed in several studies. Using mice deficient in MyD88, Yamawaki *et al.* found that LPS-induced STAT3 phosphorylation in different organs were predominantly mediated through MyD88 [46], suggesting the requirement of TLR4 signaling for efficient STAT3 activation. In our study, we noticed LPS-dependent STAT3 phosphorylation initiated at 2 h and prolonged at longer time to facilitate its role as a transcription factor for down-stream signaling. Blocking pSTAT3 using Static greatly attenuated the down-stream LPS effects for TNF-α production, demonstrating synergistic action of STAT3 for LPS-dependent TNF-α production in macrophages. Furthermore, we noticed a remarkable feature when macrophages were pre-treated with rmMFG-E8 and then stimulated with LPS, the LPS effect for STAT3 activation was markedly reduced in a time-dependent manner. We speculated this phenomenon might be due to the over production of down-stream negative regulator during rmMFG-E8 pre-treatment which in turn modulated the LPS-dependent TLR4 signaling for STAT3 activation, and finally attenuated the synergistic effects of STAT3 for TNF-α production. TLR4 signaling is under control of several negative regulators, which are mostly activated by LPS. Modulation of negative regulators either by over-expression or gene silencing may cause altered TLR4 signaling [10], [47]. SOCS families of proteins that are induced by a wide range of stimuli serve as a negative regulator of a number of signaling pathways, e.g., JAK/STAT, TLR, integrin, insulin. Induced by various cytokines, growth factors and hormones, STAT proteins serve as a pivotal transcription factor for SOCS gene expression which in turn exerts negative regulation on STAT signaling. Recent studies clearly revealed that how SOCS1 interacts with the intracellular TLR4 adaptor molecules, as well as the p65 of NF-κB, rendering them for ubiquitination and proteosomal degradation [27]–[29]. Our findings demonstrated a new insight where MFG-E8-treated macrophages clearly induced SOCS3 expression, which in turn interacted with the NF-κB p65 protein and interfered with the TLR4 signaling as a potential negative regulator (Fig. 7).

Although several therapeutic strategies are being proposed for controlling sepsis induced endotoxemia, none have been found to be totally effective. Conventional therapies for endotoxemia focus on suppression and control of inflammation using corticosteroids, and immune-modulating drugs [48], [49]. Recently developed novel cytokine antagonist therapies targeting TNF-α and IL-6 have been found to be quite effective in certain types of endotoxemia [37]. Our findings of MFG-E8-mediated attenuation of LPS-induced TNF-α production implicates an outstanding therapeutic potential for ameliorating inflammatory consequences in various diseases, hence improving the “quality of life”.

In summary, we demonstrated the MFG-E8-mediated inhibition of LPS-induced TNF-α release in macrophages through the mechanism involving STAT3-dependent SOCS3 activation, which in turn highlighted the new approach of involving the TLR4 negative regulatory system for generating the direct beneficial effects of MFG-E8 on innate-immune systems.
